# The effect of diet-induced weight loss on circulating homocysteine levels in people with obesity and type 2 diabetes

**DOI:** 10.1186/s12937-023-00908-y

**Published:** 2024-01-03

**Authors:** Meryem Al Fatly, Monique T. Mulder, Jeanine Roeters van Lennep, Henk J. Blom, Kirsten A.C. Berk

**Affiliations:** 1 Department of Internal Medicine, section of Pharmacology, Vascular and Metabolic Diseases, Erasmus University Medical Center, Rotterdam, the Netherlands; 2 Laboratory of Genetic Metabolic Diseases, Department of Clinical Genetics, Center of lysosomal and metabolic disorders, Erasmus University Medical Center, Rotterdam, The Netherlands; 3 Department of Internal Medicine, section of Dietetics, Erasmus University Medical Center, Rotterdam, The Netherlands

**Keywords:** Homocysteine, Weight loss, Calorie restricted diet, Type 2 Diabetes, Obesity, Cardiovascular Risk

## Abstract

**Background/aims:**

Having type 2 diabetes (T2D) in combination with being overweight results in an additional increase in cardiovascular disease (CVD) risk. In addition, T2D and obesity are associated with increased levels of total homocysteine (tHcy), possibly contributing to the CVD risk. Weight loss dieting has positive effects on several CVD risk factors, but whether it affects tHcy remains unclear. Therefore, the aim of this study was to determine the effect of a calorie restricted diet on tHcy in overweight people with T2D.

**Methods:**

In this post-hoc analysis of the POWER study, adults with T2D and a BMI greater than 27 kg/m² were included from the outpatient diabetes clinic of the Erasmus Medical Center, Rotterdam. The patients were subjected to a very low-calorie diet with fortified meal replacements for 20 weeks. Before and after this intervention, blood samples were collected to measure tHcy and other CVD risk factors like glycaemic and lipid parameters.

**Results:**

161 overweight participants with T2D were included, with a mean age of 54 years (range 26–74), mean weight of 104.6 ± 19.9 kg and mean HbA1c of 62.7 ± 14.3 mmol/mol. At baseline, men displayed higher tHcy than women, and tHcy level was positively correlated with body weight and triglyceride levels, while it was negatively correlated with renal function and HDL cholesterol. During the intervention, bodyweight was reduced by a mean of 9.7% (from 104.6 ± 19.9 to 94.5 ± 18.1 kg p < 0.001), and all measured glycaemic and lipid blood parameters improved significantly. However, tHcy remained unchanged (from 12.1 ± 4.1 to 12.1 ± 4.2 umol/L, p = 0.880). The change in tHcy during the intervention was negatively associated with the change in weight and BMI (p = 0.01 and p = 0.008, respectively). People who lost < 10 kg (n = 92) had a mean tHcy change of -0.47 umol/L, while people who lost more than ≥ 10 kg (n = 69) had a mean tHcy change of 0.60 umol/L (p = 0.021).

**Conclusion:**

In conclusion, our data show that a calorie restricted diet does not affect tHcy in people with T2D and obesity, despite the use of meal replacements fortified with folic acid and vitamin B12. Our data showed a negative correlation between change in tHcy levels and weight loss, suggesting that people who lost more weight (> 10 kg) showed an increase in tHcy. Future studies should explore the potential increase in tHcy induced by weight loss dieting and target the question if tHcy reduction strategies during weight loss could be clinically beneficial.

## Background/aim

People with type 2 diabetes (T2D) have an increased risk of cardiovascular diseases (CVD) [1]. Having obesity in addition to T2D further increases CVD risk [2] and reduces the life expectancy of people with T2D [3].

Elevated total homocysteine levels (tHcy) have also been associated with an increased risk of CVD [4–6]. Homocysteine is an intermediate of the methylation/remethylation cycle, which requires vitamin B12 and folic acid as cofactors. Homocysteine is degraded via the transsulfuration pathway into cysteine, requiring vitamin B6 as cofactor [7]. Normally, homocysteine remains low in the bloodstream. Circulating homocysteine levels higher than 15 umol/L are associated with arterial and venous occlusive diseases [8]. In people with diabetes, high circulating levels of Hcy have been reported [9], especially in combination with nephropathy [4]. Elevated levels of tHcy have been associated with risk of atherosclerosis, CVD and mortality in diabetes [10, 11]. Data on tHcy lowering therapies indicate they reduce DM microvascular complications, however, their effects on CVD risk reduction are conflicting [12].

Weight loss is one of the preferred therapies for people with obesity related T2D. Calorie restricted dieting is an effective method for weight loss [13]. Positive effects of weight loss on CVD risk factors, including circulating concentrations of total- and low-density lipoprotein (LDL)-cholesterol, have been previously reported [14, 15]. Moreover, a large enough and sustained weight reduction reduces cardiovascular complications in T2D [16].

Several pathways have been described for explaining the effects of weight loss on CVD risk, with the known CVD risk factors (LDL cholesterol, glucose, blood pressure) as the main mediating factors [17]. Increased tHcy is more common in obese than healthy-weight individuals, and even more in people with obesity-related T2D [18]. However, whether tHcy is a mediating factor between weight loss and reduction of CVD risk is not known. Weight loss through a calorie-restricted diet in obese people without diabetes did not lead to a change in Hcy levels [19]. Whether weight loss affects tHcy levels in people with obesity related T2D is currently unknown. The aim of this study is to assess the effect of weight loss induced by a very low-calorie diet on tHcy levels in obese patients with T2D.

## Material/methods

**Fig. 1:**
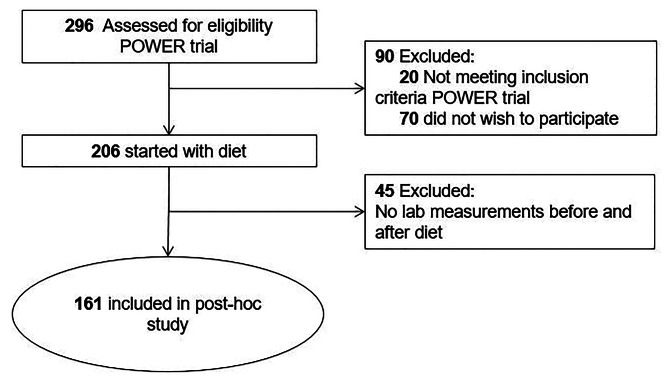
Flow-chart

In this post-hoc analysis, a prospective observational study, people recruited for the Prevention Of WEight Regain (POWER) trial were enrolled (trial registration no. NTR2264) [20]. See Fig. 1 for the flow chart of the current post-hoc study. Participants were recruited at the outpatient diabetes clinic of the Erasmus Medical Centre, Rotterdam, The Netherlands between March 2010 and April 2015. This research has been approved by The Medical Ethics Committee of the Erasmus Medical Center of Rotterdam, the Netherlands, (reference number MEC-2009-143/NL26508.078.09) in accordance with the Helsinki declaration. All participants have provided written informed consent.

### Study population

As previously described [20], this study included adults with T2D and a Body Mass Index (BMI) ≥ 27 kg/m^2^, from the outpatient diabetic clinic of the Erasmus Medical Center of Rotterdam. Exclusion criteria were pregnancy, lactation, severe psychiatric problems, significant cardiac arrhythmias, unstable angina, decompensated congestive heart failure, major organ system failure, untreated hypothyroidism, and end-stage renal disease, and a myocardial infarction, cerebrovascular accident or major surgery during the previous 3 months.

No power calculation was performed since this is a post-hoc analysis with a fixed number of participants.

### Diet intervention

The patients were subjected to a very low-calorie diet of 750 kcal per day for 8 weeks, consisting of 67 g carbohydrates, 54 g protein and 32 g fat (of which 16 g were monounsaturated fatty acids) and micronutrients following the national nutritional guidelines recommendation. This included a daily amount of 414 ug folic acid, 2.62 mg vitamin B6 and 3.21 ug vitamin B12. Diabetes-specific meal replacements (Glucerna SR®) were used 2 times a day, for breakfast and lunch. Following the very low-calorie diet, the patients were subjected to a low-calorie diet of 1100–1300 kcal per day for another 12 weeks.

### Data collection and measurement of tHcy

Before and after the dietary intervention of 20 weeks, the participants’ characteristics, including age, sex, ethnicity (Caucasian or non-Caucasian), weight, height, BMI and waist circumference were registered. Plasma tHcy, glycaemic and lipid blood parameters and systolic blood pressure were measured. Furthermore, data on insulin usage, the presence of micro- and macrovascular complications, smoking status and alcohol usage were collected.

To measure tHcy before and after intervention, blood samples were collected after an overnight fast and stored for 3–4 h in the refrigerator, after which they were centrifuged, and plasma was stored at -80℃ until analysis. Plasma tHcy was measured in citrate plasma after reduction by DTT (dithiothreitol) by LC-MS/MS (Waters Acquity UPLC coupled to Quatro Primier XE). The glycaemic and lipid parameters, including HbA1c, fasting glucose, total, HDL and LDL cholesterol, triglycerides and Apolipoprotein B (ApoB) were analysed using standard lab techniques. Plasma lipoprotein(a) was measured using a particle-enhanced immunoturbidimetric assay (Diagnostic System #171,399,910,930; DiaSys Diagnostic System, GmbH, Holzheim, Germany).

### Statistical analysis

The normality and homogeneity of the data was tested using the Shapiro-Wilk test and Kolmogorov-Smirrnov test. We report SD as a dispersion measure of variables with normal distribution, and the IQR of variables with non-normal distribution. The statistical significance of the change of the variables before and after intervention was tested using the paired t-test or the related-Samples Wilcoxon Signed Rank Test. Linear regression (LR) was applied to study the relation between patient characteristics, including weight (change), and tHcy (change). Regression plots showed that requirements of LR were fulfilled. Two-sided *P*-values < 0.05 were considered statistically significant. All analyses were carried out using IBM SPSS statistics version 26.

## Results

**Fig. 2:**
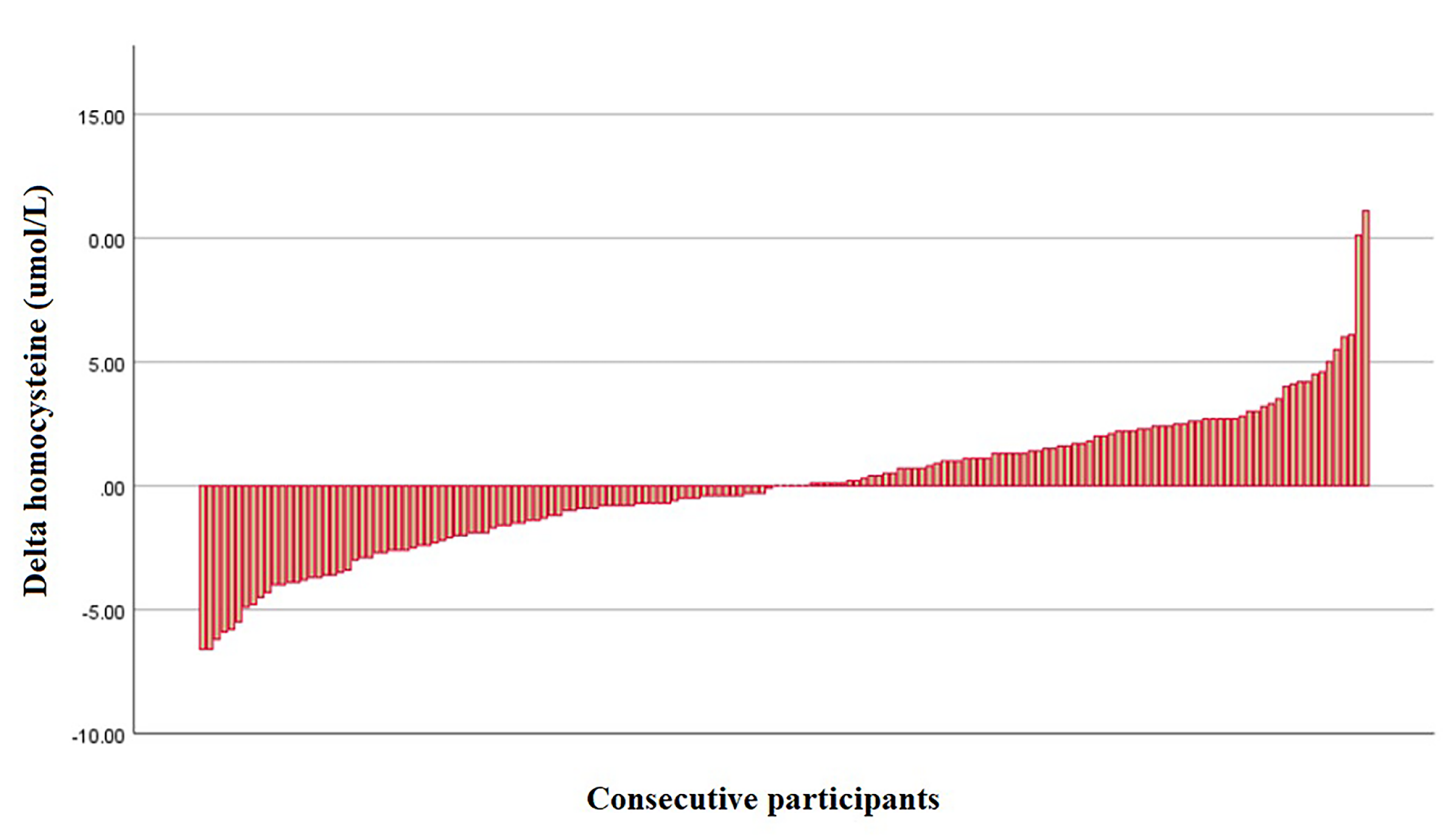
Change in homocysteine after weight-reducing dietary intervention. Change in plasma homocysteine levels during 20 weeks of weight loss diet in individual participants. Participants were arranged according to absolute change in plasma homocysteine levels

Baseline characteristics and the change in glycaemic and lipid blood parameters upon weight loss have been partly described previously [21]. This post-hoc analysis included 161 participants with a mean age of 54 years (range 26–74). Of the participants, 44.1% were male and 55.7% were Caucasian. In Table 1, we present the baseline characteristics and results after intervention. During the diet, the mean weight was reduced by 9.7% (from 104.6 ± 19.9 to 94.5 ± 18.1 p < 0.001). Likewise, the mean BMI was reduced by 9.6% (from 36.4 ± 5.8 to 32.9 ± 5.4 kg/m², p < 0.001). The measured glycaemic parameters HbA1c and fasting glucose decreased significantly during the diet intervention (p < 0.001). All lipid levels (total cholesterol, HDL cholesterol, LDL cholesterol, triglycerides and ApoB) improved significantly (p < 0.001). The systolic blood pressure did not change over time (p = 0.405). Plasma tHcy remained unchanged (from 12.1 ± 4.1 to 12.1 ± 4.2 umol/L, p = 0.880). Figure 2 presents the change in tHcy levels during the diet per participant.

Men had 2.4 umol/L higher mean baseline tHcy than women (13.5 versus 11.1; p < 0.001). Patients with Caucasian ethnicity had 1.2 umol/L higher mean values than non-Caucasians (12.4 versus 11.6; p = 0.058). Baseline body weight (mean + 0.05 umol/L per kg; p = 0.002), waist circumference (mean + 0.07 umol/L per cm; p = 0.007) and total triglyceride levels (mean + 0.79 umol/L per mmol/L; p < 0.001) were positively associated with tHcy, whereas HDL cholesterol (mean − 3.4 umol/L per mmol/L; p < 0.001) and glomerular filtration rate (GFR) (mean − 0.11 umol/L per mL/min; p < 0.001) were negatively associated.

**Fig. 3:**
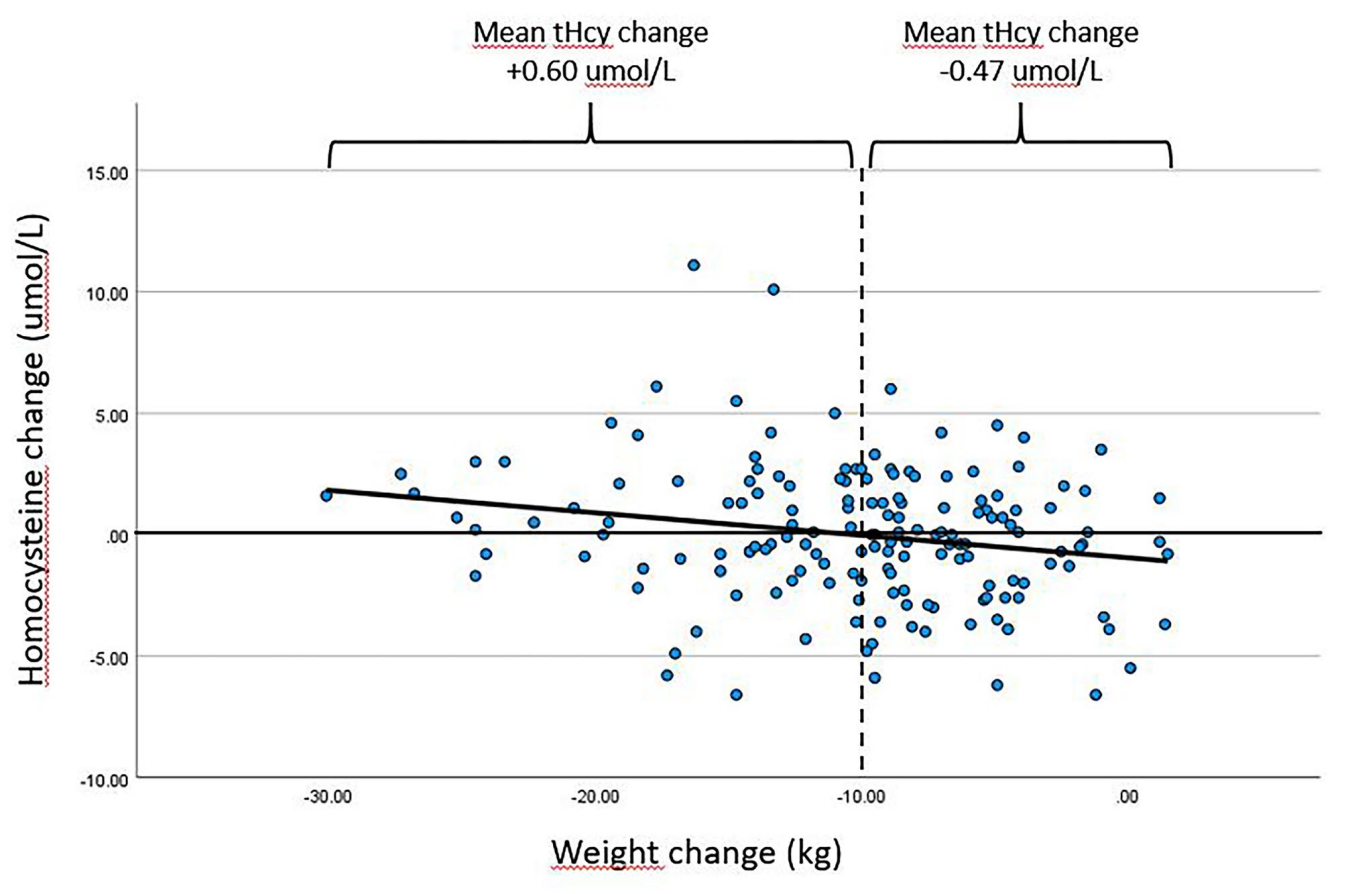
Association between change in homocysteine and change in weight during the diet intervention. This figure depicts the association between weight change (kg) and homocysteine change (umol/L), were people who lost less than 10 kg (n = 92) had a mean tHcy change of -0.47 umol/L, while people who lost more than 10 kg (n = 69) had a mean tHcy change of 0.60 umol/L (p = 0.021). tHcy = total homocysteine level

The changes in body weight and BMI were negatively correlated with a change in tHcy (Fig. 3). After adjustment for baseline tHcy (i.e. for subjects with the same baseline tHcy),1 kg extra weight reduction was associated with an extra mean tHcy change of -0.12 umol/l (*p*-value < 0.001). Factually, a weight reduction > 10 kg was related with a mean increase in tHcy levels. People who lost less than 10 kg (n = 92) had a mean tHcy change of -0.47 umol/L, while people who lost more than 10 kg (n = 69) had a mean tHcy change of 0.60 umol/L (p = 0.021). An extra one-unit BMI reduction was associated with an extra mean tHcy change of -0.34 umol/l (*p*-value < 0.001). All these relations persisted after further adjustment for sex, age, and baseline renal function (GFR), triglycerides, HDL and fasting insulin.

**Table 1:** Patient characteristics (n = 161) before and after intervention

Variables	Before intervention	After intervention	*P*-value
Age (y, range)	54 (26–74)		
Sex (% male)	44.1		
Ethnicity (% Caucasian)	73 (55.7)		
Homocysteine (umol/L)	12.1 ± 4.1	12.1 ± 4.2	0.880
Weight (kg):	104.6 ± 19.9	94.5 ± 18.1	< 0.001
BMI (kg/m²)	36.4 ± 5.8	32.9 ± 5.4	< 0.001
Waist circumference (cm)	118.7 ± 13.0	110.3 ± 12.4	< 0.001
HbA1c (mmol/mol)	62.7 ± 14.3	55.2 ± 15.6	< 0.001
Fasting glucose (mmol/L)	9.1 ± 2.8	8.2 ± 3.0	< 0.001
Total cholesterol (mmol/L)	4.6 (2.9–9.3)	4.3 (2.2–7.5)	< 0.001
HDL cholesterol (mmol/L)	1.2 (0.5–2.5)	1.2 (0.5–2.4)	< 0.001
LDL cholesterol (mmol/L)	2.7 (1.0-6.4)	2.4 (0.5–5.7)	< 0.001
Triglycerides (mmol/L)	2.5 (0.4–25.2)	1.8 (0.5–13.4)	< 0.001
ApoB (g/L)	0.9 (0.4–2.1)	0.8 (0.4–1.4)	< 0.001
Lp(a) (mg/dL)	49.7 (0.0-246.1)	60.1 (0.0-279.7)	< 0.001
Systolic blood pressure (mmHg)	140.4 ± 20.1	140.6 ± 21.0	0.405
GFR (mL/min)	75.7 ± 17.8		
Microvascular complications *n* (%)	51 (44.3)		
Macrovascular complications *n* (%)	36 (27.5)		
Insuline users *n* (%)	81 (62.3)		
Smokers *n* (%)	19 (15.3)	16 (17.2)	0.250
Alcohol users *n* (%)	33 (26.4)	19 (20.4)	0.549

*Data are mean ± SD, or median (IQR)*

N = number of patients, Y = years, BMI = body Mass Index, HDL = high density lipoprotein, LDL = low density lipoprotein, lp = lipoprotein, SD = standard deviation, IQR = interquartile range

## Discussion

Our data show that although weight loss through a calorie restricted diet induces beneficial effects on CVD risk factors such as circulating lipid and glycaemic parameters in people with T2D and obesity, it did not affect tHcy levels. Remarkably, roughly half of the participants displayed an increase, and half experienced a decrease in tHcy upon weight loss dieting, where change in tHcy level was negatively correlated with change in weight. People who lost less than 10 kg experienced a mean tHcy decrease, while people who lost more than 10 kg experienced a mean tHcy increase. At baseline, elevated tHcy level was positively correlated with obesity, Caucasian ethnicity, male sex and increased total plasma triglycerides and negatively correlated with HDL-cholesterol and renal function, which is in line with literature [5, 12, 18, 22].

Our primary finding that overall tHcy levels are not affected by diet-induced weight loss in people with obesity-related DM, has previously also been shown by studies among obese people, without diabetes [19, 22]. With this, tHcy does not appear to be a mediating factor in the beneficial effect of weight loss on cardiovascular complications in patients with T2D.

Nonetheless, our finding that the difference in effect of an energy-restricted diet on tHcy levels appears to depend on the degree of weight loss is intriguing. People who lost more weight (> 10 kg) increased in tHcy. This seems to contradict the post hoc study of the Look AHEAD trial [16], which actually saw a reduction in cardiovascular risk in people who lost more than 10 kg and were able to maintain it. One could argue that the increase in tHcy in this group may counteract the effect of weight loss on CVD, with which the effects of weight loss with tHcy lowering therapy might be even stronger. However, the evidence for this is lacking.

One could hypothesize that the people who lost more weight were more compliant with using the meal replacements, which were enriched with vitamins and minerals. The diet-specific meal replacements used in our study included a daily intake of 414 ug of folic acid and 3.21 ug of vitamin B12. Folic acid and vitamin B12 supplementation have been shown to lower tHcy concentrations [5, 22, 23]. A Cochrane study on the effect of weight loss [5] showed that in 71,422 participants with or without cardiovascular disease, a weight reducing diet with folic acid supplementation did not affect tHcy level, while a weight reducing diet without folic acid supplement caused a significant increase in tHcy level, which is in line with our overall findings. This is not in line with our hypothesis that people who were more compliant and potentially ingested more B12 and folic acid increased more in tHcy. However, we have no data on B12 and folate status, or its change during diet, so no conclusions can be drawn about the effect of supplementation. Metzner et al. [24] found that a weight reducing diet with or without meal replacement did not affect tHcy levels in 87 overweight women without T2D. However, there was a trend toward a decrease in tHcy in the group that used meal replacements, accompanied by a significant increase in folate levels. This group lost only 6 kg in 12 weeks, and thus is similar to our subgroup that lost less than 10 kg, with which the results on tHcy agree. This study did not examine the association of tHcy change with change in weight.

Associations of other lifestyle factors with homocysteine levels have also been described [25]: smoking cessation, lower coffee consumption, lower alcohol consumption, higher intake of fruits and vegetables and more aerobic exercise have been linked to lower homocysteine levels. Our intervention may have contributed to lower alcohol intake, higher fruit and vegetable intake and more aerobic exercise, and thus to lower homocysteine levels, making our finding of constant or even increasing homocysteine levels even more puzzling.

Another hypothesis is that the effect of a weight-reducing diet on tHcy levels may have been influenced by renal function. Earlier, tHcy levels have been associated with renal function markers [12]. Indeed, we observe a strong negative correlation at baseline between renal function marker (GFR) and tHcy. Since renal function has been shown to improve during weight loss dieting [26], this could have influenced the effect of weight loss on tHcy levels. Unfortunately, we do not have follow-up data on renal function in this cohort.

This study has some limitations. Firstly, no data was collected on vitamin B12 and folic acid levels at baseline and after the intervention; this hampers the evaluation of blood folic acid and B12 levels on tHcy level during weight loss dieting. Moreover, no follow-up data on renal function was available. Also, we did not collect data on body composition, such as lean body mass or fat-free mass, which influenced tHcy levels in a previous study [27]. Future studies should include these measurements prospectively. Due to the post-hoc nature of the current study, we did not perform a power analysis [28]. However, taken the small, not clinically relevant difference in Hcy, with a reasonably tight confidence interval, it is not expected that a larger study would have led to a significant clinically relevant result. Strengths of this study include the relatively large sample size, an intervention period of 20 weeks, a well-defined diet and the extensive phenotyping of the participants.

In conclusion, diet-induced weight loss showed no overall significant effect on tHcy levels in people with T2D patients and overweight. Our data showed a negative correlation between change in tHcy levels and weight loss, suggesting that people who lost more weight (> 10 kg) showed an increase in tHcy. Future studies should explore the potential increase in tHcy level induced by weight loss dieting, especially interventions inducing large weight loss, and target the question whether tHcy reducing strategies, i.e. supplementation of vitamin B6, B12 and folic acid, during weight loss could be clinically beneficial.

## Data Availability

The datasets used and/or analysed during the current study are available from the corresponding author on reasonable request.

## Abbreviations

CVD: Cardiovascular disease
GFR: Glomerular filtration rate
LDL: Low-density lipoprotein
T2D: Type 2 diabetes
tHcy: Total homocysteine levels

## References

[1] Barr EL, Zimmet PZ, Welborn TA, Jolley D, Magliano DJ, Dunstan DW (2007). Risk of cardiovascular and all-cause mortality in individuals with Diabetes Mellitus, impaired fasting glucose, and impaired glucose tolerance: the Australian Diabetes, obesity, and Lifestyle Study (AusDiab). Circulation.

[2] Piché ME, Tchernof A, Després JP (2020). Obesity phenotypes, Diabetes, and Cardiovascular Diseases. Circ Res.

[3] Grover SA, Kaouache M, Rempel P, Joseph L, Dawes M, Lau DC (2015). Years of life lost and healthy life-years lost from Diabetes and Cardiovascular Disease in overweight and obese people: a modelling study. Lancet Diabetes Endocrinol.

[4] Audelin MC, Genest J (2001). Jr. Homocysteine and Cardiovascular Disease in Diabetes Mellitus. Atherosclerosis.

[5] Martí-Carvajal AJ, Solà I, Lathyris D, Dayer M (2017). Homocysteine-lowering interventions for preventing cardiovascular events. Cochrane Database Syst Rev.

[6] Yuan S, Mason AM, Carter P, Burgess S, Larsson SC (2021). Homocysteine, B vitamins, and Cardiovascular Disease: a mendelian randomization study. BMC Med.

[7] Cm P, Homocysteine. Cleveland Clinic; 2023 [updated 2023.

[8] Refsum H, Ueland PM, Nygård O, Vollset SE (1998). Homocysteine and Cardiovascular Disease. Annu Rev Med.

[9] Mursleen MT, Riaz S (2017). Implication of homocysteine in Diabetes and impact of folate and vitamin B12 in diabetic population. Diabetes Metab Syndr.

[10] Soedamah-Muthu SS, Chaturvedi N, Teerlink T, Idzior-Walus B, Fuller JH, Stehouwer CD (2005). Plasma homocysteine and microvascular and macrovascular Complications in type 1 Diabetes: a cross-sectional nested case-control study. J Intern Med.

[11] Lu J, Chen K, Chen W, Liu C, Jiang X, Ma Z (2022). Association of Serum Homocysteine with Cardiovascular and all-cause mortality in adults with Diabetes: a prospective cohort study. Oxid Med Cell Longev.

[12] Muzurović E, Kraljević I, Solak M, Dragnić S, Mikhailidis DP (2021). Homocysteine and Diabetes: role in macrovascular and microvascular Complications. J Diabetes Complications.

[13] American Diabetes A (2020). 8. Obesity management for the treatment of type 2 Diabetes: standards of Medical Care in Diabetes—2021. Diabetes Care.

[14] Harder H, Dinesen B, Astrup A (2004). The effect of a rapid weight loss on lipid profile and glycemic control in obese type 2 diabetic patients. Int J Obes Relat Metab Disord.

[15] Dhindsa P, Scott AR, Donnelly R (2003). Metabolic and cardiovascular effects of very-low-calorie diet therapy in obese patients with type 2 Diabetes in secondary failure: outcomes after 1 year. Diabet Med.

[16] Look ARG, Gregg EW, Jakicic JM, Blackburn G, Bloomquist P, Bray GA (2016). Association of the magnitude of weight loss and changes in physical fitness with long-term Cardiovascular Disease outcomes in overweight or obese people with type 2 Diabetes: a post-hoc analysis of the look AHEAD randomised clinical trial. Lancet Diabetes Endocrinol.

[17] Diseases C, Lu Y, Hajifathalian K, Ezzati M, Woodward M, Rimm EB, Global Burden of Metabolic Risk Factors for Chronic (2014). Metabolic mediators of the effects of body-mass index, overweight, and obesity on coronary Heart Disease and Stroke: a pooled analysis of 97 prospective cohorts with 1·8 million participants. Lancet.

[18] Karatela RA, Sainani GS (2009). Plasma homocysteine in obese, overweight and normal weight hypertensives and normotensives. Indian Heart J.

[19] Persil-Ozkan O, Yigit E, Yigit Z (2019). Does weight loss affect the parameters that are metabolically related to Cardiovascular Diseases?. Saudi Med J.

[20] Berk KA, Buijks H, Ozcan B, Van’t Spijker A, Busschbach JJ, Sijbrands EJ (2012). The Prevention of WEight regain in Diabetes type 2 (POWER) study: the effectiveness of adding a combined psychological intervention to a very low calorie diet, design and pilot data of a randomized controlled trial. BMC Public Health.

[21] Berk KA, Yahya R, Verhoeven AJM, Touw J, Leijten FP, van Rossum EF (2017). Effect of diet-induced weight loss on lipoprotein(a) levels in obese individuals with and without type 2 Diabetes. Diabetologia.

[22] Sudchada P, Saokaew S, Sridetch S, Incampa S, Jaiyen S, Khaithong W (2012). Effect of folic acid supplementation on plasma total homocysteine levels and glycemic control in patients with type 2 Diabetes: a systematic review and meta-analysis. Diabetes Res Clin Pract.

[23] van Oort FV, Melse-Boonstra A, Brouwer IA, Clarke R, West CE, Katan MB (2003). Folic acid and reduction of plasma homocysteine concentrations in older adults: a dose-response study. Am J Clin Nutr.

[24] Metzner CE, Folberth-Vögele A, Bitterlich N, Lemperle M, Schäfer S, Alteheld B (2011). Effect of a conventional energy-restricted modified diet with or without meal replacement on weight loss and cardiometabolic risk profile in overweight women. Nutr Metab (Lond).

[25] Panagiotakos DB, Pitsavos C, Zeimbekis A, Chrysohoou C, Stefanadis C (2005). The association between lifestyle-related factors and plasma homocysteine levels in healthy individuals from the ATTICA Study. Int J Cardiol.

[26] Tirosh A, Golan R, Harman-Boehm I, Henkin Y, Schwarzfuchs D, Rudich A (2013). Renal function following three distinct weight loss dietary strategies during 2 years of a randomized controlled trial. Diabetes Care.

[27] Park SB, Georgiades A (2013). Changes in body composition predict homocysteine changes and hyperhomocysteinemia in Korea. J Korean Med Sci.

[28] Althouse AD (2021). Post Hoc Power: not empowering, just misleading. J Surg Res.

